# Cochlear amyloid beta 42–mediated hearing loss: A marker of preclinical Alzheimer’s disease

**DOI:** 10.4103/NRR.NRR-D-25-01128

**Published:** 2026-01-02

**Authors:** Dheyaa Al-Sallami, Shelley Tischkau, Raheem F. H. Al Aameri, Leonard P. Rybak

**Affiliations:** Department of Pharmacology, Southern Illinois University School of Medicine, Springfield, IL, USA

**The emerging connection:** As populations age, the link between age-related hearing loss (ARHL) and Alzheimer’s disease (AD) is emerging as one of the most urgent public health challenges of our time. Recent research analyzing 2946 community-dwelling adults revealed that hearing loss may contribute to nearly one-third of all new dementia cases — significantly higher than previous estimates (Ishak et al., 2025). This epidemiological evidence suggests a mechanistic rather than coincidental relationship. Longitudinal studies show hearing loss precedes cognitive decline by 5–10 years. The implications are demographically profound. The yearly costs related to dementia are over $1.3 trillion, and untreated hearing loss adds another $133 billion in lost productivity. As a result, it has become essential to clarify the mechanistic link between these disorders. ARHL affects approximately one-third of adults over 65 years and rises to 75% in those over 80 years. In contrast, AD remains the leading cause of dementia, accounting for 60%–80% of all cases (Livingston et al., 2020).

Classic explanations linking ARHL and AD are sensory deprivation, cognitive overload, and social isolation. These factors are contributory but cannot completely explain the strong temporal precedence and biological plausibility of hearing loss as a precursor to neurodegeneration. The sensory deprivation hypothesis posits that decreased auditory input causes compensatory cortical reorganization, which in turn disrupts cognitive processes. Conversely, the cognitive overload theory suggests that processing degraded auditory signals exhausts cognitive resources. However, these processes, in isolation, cannot account for the consistent time gap between the onset of hearing loss and cognitive impairment.

The identification of molecular pathways common to both ARHL and AD, notably involving amyloid-beta (Aβ) pathology, has created new possibilities for investigating this link and creating targeted therapeutic approaches. Emerging evidence reveals the cochlea as an early location of Aβ accumulation, thus identifying this as a potential biomarker for impending AD.

**The cochlear aqueduct — a direct conduit:** The cochlear aqueduct (CA) provides the anatomical conduit for transport of cerebrospinal fluid (CSF) between the brain and the inner ear. This thin bony duct, having an average diameter of 138 ± 58 µm in humans and 122 ± 5 µm in mice, allows regulated exchange between CSF and perilymph while restricting bulk fluid movements. It facilitates pressure equilibration between the intracranial space and inner ear (Mathiesen et al., 2023). Under physiological circumstances, this anatomical structure acts as a protective mechanism for the inner ear by shielding it from fluctuations in CSF pressure and maintaining the fragile ionic balance necessary for auditory signal transduction.

The distinctive anatomy of CA makes it especially susceptible to pathological infiltration. In contrast to the blood–labyrinth barrier (BLB), which actively limits molecular transfer, the aqueduct allows selective exchange of small molecules and proteins between the inner ear and the central nervous system.

In Vglut3^–/–^ mice, dynamic contrast-enhanced MRI showed that CSF-injected tracers reach cochlear perilymph via the CA within minutes (Mathiesen et al., 2023). In AppNL-G-F/NL-G-F knock-in mice, proteomic analysis demonstrated that the CA functions as a passive, size-selective conduit permitting small molecules (< 5 kDa) to equilibrate between CSF and perilymph while excluding larger proteins (Fukuda et al., 2024). In pneumococcal meningitis, bacteria invade the cochlea via the CA within 12–15 hours post-infection, leading to acute labyrinthitis and rapid hearing loss.

These findings confirm the CA as a viable pathway for brain–inner ear communication. Because Aβ_42_ monomers (**~**4.5 kDa) fall below the aqueduct’s size cutoff, it may allow Aβ_42_ to move from the CNS into cochlear compartments in early AD, circumventing the BLB.

**Human evidence — Aβ**_**42**_ **and Tau in perilymph:** Innovative human research has identified AD-associated biomarkers, such as Aβ_42_, Aβ_40_, and tau proteins, in the cochlear perilymph obtained during cochlear implantation procedures (Walia et al., 2024). While direct human evidence is confined so far to this first-ever study (*n* = 25 cochlear implant patients), it presents important proof-of-concept that perilymph contains human biomarkers linked to AD. Marker concentrations were approximately 10 times lower compared to CSF, with significant correlations for patient age and cognitive ability. Beyond serving as systemic indicators, perilymph biomarkers showed a connection with both concentration gradients as well as cognitive ability, suggesting active transport as well as localized metabolism within cochlear tissue. Notably, the proportion of perilymphatic Aβ_42_ relative to Aβ_40_ inversely correlated with cognitive ability, as observed in the CSF, thereby validating the promise of cochlear biomarkers.

The occurrence of phosphorylated tau in human perilymph presents direct evidence of tau pathology participation in cochlear impairment. In central nervous system scenarios, tau hyperphosphorylation interferes with synaptic vesicle movement and transmitter release. Conversely, cochlear tau pathology likely disrupts synaptic transmission between inner hair cells and auditory nerve fibers, as the cause of the early hearing impairments before cognitive symptomatology. Co-occurrence of both Aβ_42_ and phosphorylated tau in perilymph implies the working together of both pathologies — Aβ_42_ perhaps inducing cellular stress, but tau pathology continues synaptic impairment, establishing auditory dysfunction before central cognitive decline.

These findings provide crucial proof-of-concept for cochlear biomarkers for early AD diagnosis. The link between perilymph biomarkers and cognitive function raises the possibility that cochlear analysis might enable earlier AD diagnosis than current methods. Cognitive deterioration usually follows hearing loss by multiple years. Thus, cochlear biomarkers could usher in a clinically noteworthy intervention time frame, subject to longitudinal prospective confirmation. Existing evidence stems from a retrospective single-center study of patients with cochlear implants (*n* = 25), and multi-centered confirmation in more heterogeneous populations with standardized minimally invasive sampling protocols is therefore required.

**Figure 1:**
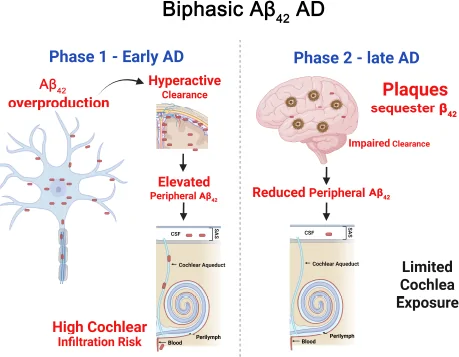
Biphasic dynamics of Aβ_42_ clearance and cochlear exposure across Alzheimer’s disease progression. This model shows two phases of Aβ_42_ clearance during early AD and their link to cochlear exposure. Phase 1 (Compensatory hyperactivity): Early AD features brain overproduction of Aβ_42_, activating hyperactive clearance mechanisms like enhanced glymphatic pathway and LRP1 blood–brain barrier transport. This raises peripheral Aβ_42_ levels (8.01 pg/mL higher in future AD patients, *P* = 0.006) that penetrate the cochlear aqueduct and blood-labyrinth barrier, seeding inner ear tissues with amyloid peptides. Phase 2 (Clearance failure): Saturation and decline of clearance proteins (neprilysin, insulin-degrading enzyme) reduce peripheral clearance. Though cochlear exposure to new Aβ_42_ drops, permanent auditory damage from phase 1 persists. The transition marks a critical therapeutic window for cochlear-targeted interventions. Created in BioRender. Al sallami, D. (2025) https://BioRender.com/eze7o4k. Aβ_42_: Amyloid-beta 42; AD: Alzheimer’s disease; CSF: cerebrospinal fluid; LRP1: low-density lipoprotein receptor-related protein 1; SAS: subarachnoid space.

**Biphasic clearance dynamics:** Early AD may exhibit unique biphasic Aβ_42_ clearance kinetics that offers important information regarding cochlear exposure patterns. During the initial phase, brain overproduction induces compensatory hyperactivity of clearance mechanisms, leading to heightened peripheral Aβ_42_ levels with the potential to seed cochlear tissues. This compensatory period represents a vulnerable window during which peripheral organs, such as the cochlea, can become exposed to pathological concentrations of amyloid peptides (**Figure 1**).

Meta-analytic work invariably shows that cognitively healthy persons who later developed AD showed significantly higher baseline plasma Aβ_42_ levels compared with those who remained cognitively normal with a weighted mean difference of 8.01 pg/mL (*P* = 0.006) (Song et al., 2011). This elevation may reflect an early imbalance in Aβ production and clearance, though the specific mechanisms remain to be fully characterized. The brain’s major clearance routes — particularly the glymphatic pathway and LRP1-mediated blood–brain barrier (BBB) transport — function to remove soluble Aβ_42_ from brain (Tarasoff-Conway et al., 2015). During sleep, the glymphatic system accelerates Aβ removal through aquaporin-4 channels on astrocytic endfeet. Concurrently, upon entering the compensatory stage, the LRP1 transport across the BBB allows the mobilization of soluble Aβ_42_ from the brain environment into the peripheral blood, leading to a temporary elevation of plasma levels.

The above-cited compensatory mechanisms are not tenable for long periods and eventually result in saturation of clearance systems, which leads to subsequent deterioration. In late-onset AD, enhanced cerebral Aβ accumulation is mainly attributed to reduced clearance of Aβ and not to increased production, thus emphasizing the inefficient clearance mechanism as an important player in the pathology. This chronic exposure down-regulates several clearance genes, notably neprilysin and insulin-degrading enzyme, and compromises peripheral clearance ability.

This proposed two-phase clearance pattern may potentially explain why the cochlea is transiently exposed to elevated Aβ_42_ levels early in cognitive decline. During the early hyperactive phase of AD, high circulating Aβ_42_ may permeate the CA and BLB, entering inner-ear structures. Because the mechanisms of Aβ_42_ clearance decrease over time, peripherally measured Aβ_42_ levels decline. Early cochlear injury, however, may persist despite lower peripheral Aβ_42_. This persistence suggests a potential therapeutic window for intervening at an early stage of AD to prevent irreversible damage. The biphasic clearance model discussed herein constitutes a theoretical construct that necessitates empirical verification via longitudinal studies assessing both central (CSF, imaging) and peripheral (perilymph, plasma) dynamics of Aβ_42_ throughout the progression of AD.

**Molecular mechanisms in cochlear pathology:** The cochlea possesses sophisticated molecular machinery for processing of the amyloid precursor protein and Aβ metabolism, including beta-secretase (BACE1) expression. BACE1 is a key enzyme for normal auditory function (Dierich et al., 2019). BACE1-null mice are profoundly deaf, with significantly increased auditory brainstem response thresholds and lower distortion product otoacoustic emissions, thus indicating that amyloid precursor protein processing is a necessary function of cochlear tissues. Receptors for entry and degradation of Aβ are found on different cochlear structures. LRP1 is highly expressed in ganglion neurons, endothelial cells, inner hair cells, and stria vascularis, whereas the RAGE receptor is found in the organ of Corti, spiral ganglion, and stria vascularis (Shi et al., 2022). These receptors are molecular targets that contribute to the passage of Aβ through the BLB and can be potential therapeutic targets.

The BLB, analogous to the BBB but lacking astrocytic endfeet, depends on tight junctions between endothelial cells and pericytes to maintain selective permeability. In inflammatory conditions, exposure to cytokines such as TNF-α and IL-6 disrupts these junctions — marked by loss of ZO-1 and occludin — and increases barrier permeability, compromising BLB function (Sekulic et al., 2023).

The cochlear pathological process of AD encompasses several integrated mechanisms at the cellular and molecular levels. Firstly, direct toxicity of Aβ acts on spiral ganglion neurons and on hair cells by protein aggregation, oxidative insult, and cellular homeostatic disruption. Secondly, tau-induced synaptic impairment results when phosphorylated tau perturbs normal synaptic transmission at inner hair cell-auditory nerve synapses. Thirdly, oxidative stress and cochlear mitochondrial impairment especially affect high-energy cochlear structures critical for hearing transduction as well as neural signal transmission. Fourthly, neuroinflammatory reactions involving macrophage activation and the release of cytokines compromise the integrity of the blood-labyrinth barrier as well as the cochlear microenvironment. Lastly, deranged ion homeostasis influences the cycling of potassium as well as the generation of the endocochlear potential, an indispensable component of cochlear amplification as well as hearing sensitivity.

**Clinical implications, limitations, and future directions:** Detection of cochlear amyloid disease contains tremendous potential for the treatment of early AD. Biomarkers based on perilymph could provide less invasive alternatives for the measurement of AD-linked cerebrospinal fluid analytes without the technical challenges or patient compliance problems. Although existing cochlear implant procedures allow perilymph sampling in well-defined patient cohorts, the development of less invasive sampling — transtympanic micro-sampling or endoscopic round-window procedures — could bring broader at-risk cohorts within the range of analysis. In addition to diagnostics, the cochlear–brain axis also identifies a new therapeutic target: localized drug delivery through the cochlear aqueduct, localized clearance of amyloid, or repair of dysfunctional cochlear clearance mechanisms could prevent irreversible neurodegeneration. Inclusion of audiometric tests, speech-in-noise recognition, and auditory brainstem responses among others in the screening protocols for AD could provide additional improvement for the diagnosis at the preclinical stage.

Several promising findings lay the groundwork for clinical translation, but additional work is needed to expand applicability. First, the bulk of evidence supporting them comes from murine models, where the diameter of the cochlear aqueduct (122 ± 5 µm) substantially differs from the human value (138 ± 58 µm), perhaps affecting molecular clearance kinetics. Additionally, the available human data apply only to the small minority receiving cochlear implants, thus limiting broader applicability. Again, the postulated biphasic clearance mechanism of Aβ_42_ remains speculative without direct human-subject experimentation.

Future studies must strive to fill these gaps identified here by conducting multi-center validation studies with different participant groups and developing standardized, minimally invasive sampling protocols. Translational studies in the future will also be critical in defining the threshold for sensitivity and specificity of detection of perilymph Aβ_42_ compared with established CSF and plasma biomarkers and in defining the best time points for detection and intervention. Each of these studies will establish the potential of cochlear measures either as complements or surrogates for the early identification and treatment of AD.

**Conclusion:** Preliminary results indicate that cochlear biomarkers could bring an innovative understanding of AD pathogenesis, but important research gaps need to be addressed before their use in the clinic. Suggested biphasic clearance model of Aβ_42_ based on an initial phase of peripheral over-exposure followed by subsequent impairment of clearance emphasizes the need for validating the dynamics of peripheral perilymph Aβ in relation to central disease. Future studies should then validate the perilymph analyte findings in larger groups of people who vary demographically; develop and standardize minimally invasive sampling procedures as well as analytical protocols; apply advanced audiometric measures, such as speech-in-noise tests and auditory brainstem responses, to risk stratification algorithms. With due circumspection and strict validation, exploitation of the cochlear–brain interface may allow the disclosure of AD pathology at the important preclinical stage of the disease, thus providing the basis for earlier, more tailored intervention and improved patient outcomes.

*Presentation at a meeting: 35*^*th*^ *Annual Trainee Research Symposium, Southern Illinois University School of Medicine, Springfield, IL, USA on April 18, 2025.*
